# Implicit Mentalizing in Patients With Schizophrenia: A Systematic Review and Meta-Analysis

**DOI:** 10.3389/fpsyg.2022.790494

**Published:** 2022-02-02

**Authors:** Timea Csulak, András Hajnal, Szabolcs Kiss, Fanni Dembrovszky, Margit Varjú-Solymár, Zoltán Sipos, Márton Aron Kovács, Márton Herold, Eszter Varga, Péter Hegyi, Tamás Tényi, Róbert Herold

**Affiliations:** ^1^Department of Psychiatry and Psychotherapy, Medical School, University of Pécs, Pécs, Hungary; ^2^Doctoral School of Clinical Neurosciences, Medical School, University of Pécs, Pécs, Hungary; ^3^Medical School, Institute for Translational Medicine, University of Pécs, Pécs, Hungary; ^4^Department of Pediatrics, Medical School, University of Pécs, Pécs, Hungary

**Keywords:** mentalizing, theory of mind, mentalization, implicit, schizophrenia, spontaneous

## Abstract

**Introduction:**

Mentalizing is a key aspect of social cognition. Several researchers assume that mentalization has two systems, an explicit one (conscious, relatively slow, flexible, verbal, inferential) and an implicit one (unconscious, automatic, fast, non-verbal, intuitive). In schizophrenia, several studies have confirmed the deficit of explicit mentalizing, but little data are available on non-explicit mentalizing. However, increasing research activity can be detected recently in implicit mentalizing. The aim of this systematic review and meta-analysis is to summarize the existing results of implicit mentalizing in schizophrenia

**Methods:**

A systematic search was performed in four major databases: MEDLINE, EMBASE, Cochrane Central Register of Controlled Trials (CENTRAL), Web of Science. Eleven publications were selected. Five studies were found to be eligible for quantitative synthesis, and 9 studies were included in qualitative synthesis.

**Results:**

The meta-analysis revealed significantly lower accuracy, slower reaction time during implicit mentalizing in patients with schizophrenia. The systematic review found different brain activation pattern, further alterations in visual scanning, cue fixation, face looking time, and difficulties in perspective taking.

**Discussion:**

Overall, in addition to the deficit of explicit mentalization, implicit mentalization performance is also affected in schizophrenia, if not to the same extent. It seems likely that some elements of implicit mentalization might be relatively unaffected (e.g., detection of intentionality), but the effectiveness is limited by certain neurocognitive deficits. These alterations in implicit mentalizing can also have potential therapeutic consequences.

**Systematic Review Registration:** https://www.crd.york.ac.uk/prospero/, identifier: CRD42021231312.

## Introduction

Mentalizing (or mentalization, theory of mind) is a key aspect of social cognition. During the processes of mentalizing we attribute mental states (intentions, beliefs, desires, emotional states) to ourselves and others, which enables us to understand and predict social behavior. Mentalizing is a highly complex ability requiring the perception, processing, and interpretation of social information. Traditionally mentalizing has been considered to develop in the early ages of life (3–6 years). However, more and more data suggest that children can intuitively attribute intentions much earlier (Apperly and Butterfill, 2009). According to these results, several researchers assume that mentalization is based on two systems, an explicit and an implicit one (Apperly and Butterfill, 2009; Butterfill and Apperly, 2013). Implicit mentalizing is supposed to be present very early, presumably from birth. It is characterized by fast and pre-reflexive non-verbal information processing, which is decoded without awareness. In contrast to this intuitive ability, the explicit form of mentalizing is inferential, relatively slow, and it relies heavily on verbal and conscious information processing. It develops parallelly with linguistic and cognitive skills (e.g., executive functions). The implicit-explicit systems are likely to persist and coexist throughout the lifespan (Apperly and Butterfill, 2009; Butterfill and Apperly, 2013; Vogeley, 2017). According to some recent hypotheses, different social neural networks are responsible for the processing of implicit lower-level information, and the explicit higher-level information (Vogeley, 2017). The former plays a role in the early, automatic detection of intentional bodily and spatial behavior. The latter is active in the late, controlled, and conscious evaluative and interpretive processing. However, not all researchers accept this sharp distinction between the implicit and explicit systems (Baillargeon et al., 2015; Scott and Baillargeon, 2017). Moreover, according to the submentalization approach, although certain behavioral elements may appear to be a process of implicit mentalization, it does not actually involve real mentalization, but rather domain-general cognitive processing (e.g., attention orientation, spatial perception, etc.) (Heyes, 2014).

Still there is no consensus either, whether implicit and explicit mentalizing rely on shared or distinct brain networks (Lieberman, 2007; Van Overwalle and Vandekerckhove, 2013). According to a recent meta-analysis of neuroimaging data the two types of processing overlap significantly, however important differences are also present (Molenberghs et al., 2016). Activation likelihood estimation revealed that the medial prefrontal cortex (mPFC), precuneus, bilateral inferior frontal gyrus (IFG), temporoparietal junction (TPJ), and temporal poles are activated both in implicit and explicit processing. At the same time explicit tasks activate the middle and superior temporal gyrus, the cingulate gyrus, and the medial frontal gyrus on the left side, while implicit tasks associate with the activation of left medial frontal gyrus, and right IFG. The meta-analytic connectivity modeling also revealed widespread overlapping co-activating areas during both explicit and implicit processing (Molenberghs et al., 2016). However, implicit mentalizing areas co-activate with bilateral middle frontal gyrus and the left superior frontal gyrus, while the explicit mentalizing regions co-activate with the right cingulate gyrus and left parietal lobule.

In the last 10–15 years an enormous number of studies dealt with the exploration of implicit mentalizing. Various paradigms have been developed, and their results suggest the existence of implicit mentalization. For example, Kovács et al. used an object detection task to investigate implicit mentalization, where participants' reaction time varied according to whether the other person's true or false belief was congruent or incongruent with the subject's belief. Based on their paradigm, the gaze times of 7-month-old infants have been shown to be influenced by their expectations, in the same way as for adults (Kovács et al., 2010). Although this paradigm was later contested by some (Phillips et al., 2015), more recent research has obtained results like Kovács et al. and found that one's own and the other's beliefs have a significant effect on reaction time (van der Wel et al., 2014; Nijhof et al., 2016; El Kaddouri et al., 2020). Interactive behavioral tasks (Buttelmann et al., 2009; Southgate et al., 2010); violation of expectation (Onishi and Baillargeon, 2005) is also frequently used paradigms in implicit mentalizing research. The anticipatory looking measures are widely used as well (Southgate et al., 2007; Schneider et al., 2012; Low and Watts, 2013), however the results are ambiguous because they have been found hardly replicable. Results from these paradigms are likely to be informative when they use ecologically relevant stimuli. In other situations, presumably participants just look back and forth without any anticipation, which can be one of the reasons for their non-replicability (Kulke et al., 2019; Kulke and Hinrichs, 2021). Moreover, a study using real-life mobile eye-tracking also failed to clearly confirm the suitability of anticipatory looking measures for implicit mentalization, which may also suggest that it is very difficult to detect (Kulke and Hinrichs, 2021). In summary, the existence of implicit mentalizing seems not to be questioned, however the results are still puzzling, and the appropriate tool for detecting implicit mentalizing is also missing.

In schizophrenia, it is now evident that social cognition is significantly affected and there is, among other things, a significant mentalizing deficit. Based on the research results, systematic reviews, and meta-analyses, it is clear that intention attribution of the patients is damaged (Sprong et al., 2007; Bora et al., 2009; Martin et al., 2014). Mentalizing impairments are characteristic both in the acute and the remission phases, and they can be detected in first-degree, clinically asymptomatic relatives (Herold et al., 2002, 2018; Bora and Pantelis, 2013; Healey et al., 2013). Mentalizing may be deficient even before the onset of the disease, may predict psychotic conversion, and often worsens before the first episode (Bora and Pantelis, 2013; Davidson et al., 2018; Tikka et al., 2020). Long-term studies of social functionality also suggest that functionality is already weaker in childhood and deteriorates markedly further in adolescence, which in turn significantly predicts impaired functionality over a 20 year period (Velthorst et al., 2017).

Imaging studies have also revealed significant abnormalities in schizophrenia. In addition to the brain volumetric abnormalities in pre-frontal and temporal areas (Benedetti et al., 2009; Herold et al., 2009; Koelkebeck et al., 2013) associated with deficient mentalization, studies using different functional imaging procedures have undoubtedly described atypical neural activation characterized by over- and underactivation in mentalizing regions (Marjoram et al., 2006). According to a meta-analysis, the mPFC, the left orbito-frontal cortex (OFC), and a small portion of the left posterior TPJ are regularly found under-activated, while over-activation was reported in the more dorsal part of the TPJ bilaterally, in the medial occipito-parietal cortex, right premotor areas, left cingulate gyrus, and lingual gyrus (Kronbichler et al., 2017). Moreover, different activation has been shown in high-risk patients in the right TPJ, right middle temporal gyrus (MTG), and left precuneus (Vucurovic et al., 2021), and also in clinically asymptomatic relatives in dorsolateral PFC, dorsomedial PFC, and right inferior frontal gyrus (Marjoram et al., 2006; Herold et al., 2018).

Despite the extensive research on mentalizing in schizophrenia, the majority of studies has been focused only on explicit mentalizing. Relatively little is known about potential alterations of implicit mentalizing. Based on the neurodevelopmental hypothesis of schizophrenia (Weinberger, 1987) we cannot exclude that the implicit mentalizing is also impaired, as early neurodevelopmental abnormalities may affect the neural networks responsible for implicit mentalizing, which in turn may influence the development of later explicit mentalizing skills. Research data suggest that the impaired early embryonic and later adolescent maturation of the PFC is likely to play a role not just in the development of behavioral, but also in cognitive symptoms of the disorder (Selemon and Zecevic, 2015). Studies on childhood onset schizophrenia emphasize the role of steeper rate of tissue loss in parietofrontal and parietotemporal areas as well (Gogtay, 2008). Recently, abnormal growth process of the cingulo-fronto-termporal module development was also reported, which affect several structures repeatedly found impaired in mentalizing studies (right IFG, triangular and opercular part; right medial orbital superior frontal gyrus; right gyrus rectus; left posterior cingulate gyrus) (Alexander-Bloch et al., 2014). These disturbed maturational trajectories may interfere with the development of mentalizing, and indeed the infant research data highlights the role of temporoparietal areas in the early development of implicit mentalizing (Kampis et al., 2015; Hyde et al., 2018; Grosse Wiesmann et al., 2020).

Beyond its theoretical aspect, it may have even a therapeutic significance (Langdon et al., 2017), as unaffected implicit mentalizing skills may represent a significant base for remediating the impaired explicit mentalizing skills. However, impaired implicit mentalizing can be a significant limit in remediation. Recently, Langdon et al. highlighted the therapeutic implication of the differential effects of implicit and explicit aspects of mentalizing, as the remediation of explicit mentalizing may require interventions to strengthen compensatory strategies, while implicit mentalizing may require a more basic approach with using techniques to improve attentional processes to support a more efficacious detection of agency signals (Langdon et al., 2017).

Nevertheless, beside the dominance of research on explicit mentalizing in schizophrenia, more and more study focus on the implicit mentalizing. Unfortunately, the results are still not equivocal, so we found it important to summarize the results of the field. The aim of this systematic review and meta-analysis is to examine the nature of possible implicit mentalizing alterations in schizophrenia compared to healthy controls.

For theoretical clarity in our meta-analysis and systematic review we included only those studies that used non-verbal tasks to indirectly measure the accuracy with automatic behavioral signs without verbal answers. We excluded those studies that measure mentalizing skills with verbal answers or with spontaneous use of mental-state language. The latter type of performance can be described with the term of spontaneous mentalizing, although some studies use the phrase as a synonym for implicit mentalizing (e.g., Horan et al., 2009). In contrast, elsewhere, this term refers to indirect measurements, when the processing of social information happens without explicit instruction, but it is measured with the spontaneous use of mental state terms (Senju, 2013; Langdon et al., 2017). According to Senju (2012), spontaneous mentalizing is differ from implicit mentalizing as it does not require the lack of conscious awareness. Moreover, it is not so obligatory processing like automatic processing, and it can be interrupted with competing tasks. Usually, spontaneous mentalizing is tested with animated geometric forms stimuli, and measured by multiple choice questions or spontaneous use of mental-state language. Our systematic review on spontaneous mentalizing in schizophrenia will be presented in a separate article.

## Methods

This systematic review and meta-analysis were reported based on PRISMA Statement (Page et al., 2021). The review protocol was registered on PROSPERO (CRD42021231312). There was no protocol deviation.

### Search Strategy

A systematic search was performed in four major databases: MEDLINE, EMBASE, Cochrane Central Register of Controlled Trials (CENTRAL), Web of Science.

The search date was 02.11.2020. The following search key was used: [(implicit) OR (spontaneous)] AND [(theory of mind) OR (mentalizing) OR (mentalization)] AND (schizophrenia). We searched in all fields/all text in every database. There were no restrictions or filters.

### Selection and Eligibility Criteria

The search results were combined in a reference manager software (EndNoteX9; Clarivate Analytics, Philadelphia, Pennsylvania). Records were screened (after automatic and manual removal of duplicates) based on title, abstract, full-text. Then the references and citations of the full text screening records were reviewed. The selection process was conducted by two independent researchers (AH, TC). Disagreements were resolved by an independent third investigator (RH). Reference lists, publication citing (Google Scholar search engine) of the included studies were screened to find additional studies.

We included case-control studies which reporting on implicit mentalization function in patients with schizophrenia. We also did not exclude studies that included schizoaffective patients, as both disorders belong to the same group of disorders, the schizophrenia spectrum disorders. The individuals of the control group were excluded if they met criteria for any psychiatric disorder. Studies which had overlapping populations were included only in the systematic review.

We included studies which measure implicit mentalization function with tasks taken unrelated to description of the paradigm or taken before questions of the paradigm (for e.g., eye movements measures, perspective taking tasks). As described in the introduction, we excluded records, which examined spontaneous mentalization and the spontaneous use of mentalization terms or used verbal answers to measure mentalizing performance.

### Data Extraction

Two independent review authors extracted the following data from each eligible studies: first author, publication year, study design, country, number of centers, studied population, gender distribution, age distribution, number of patients; accuracy (in percentage), reaction time (in ms), mentalizing cue looking percentage, fixation duration, face looking percentage. If the data was plotted on a bar graph, GetData Graph Digitizer was used to extract the data. We contacted the authors in case of missing data, and the data received were used during the processing. Disagreements were resolved by an independent third investigator.

### Risk of Bias Assessment

The “Quality In Prognosis Studies” (QUIPS) tool (Hayden et al., 2013) was used based on the recommendations of The Cochrane Prognosis Methods Group (PMG) by two researchers. Any disagreement was resolved by a third reviewer.

### Statistical Analysis

For continuous variables standardized mean difference (SMD) with 95% Confidence Intervals were calculated and since we had one study with sample size <20 we decided to use Hedges method. A *p* < 0.05 was considered statistically significant difference. Random effects model was used to calculate the overall estimates using the DerSimonian-Laird (DerSimonian and Laird, 1986) method. The results of the meta–analyses are presented on forest plots.

Heterogeneity was tested using Cochrane's *Q* and the *I*^2^ statistics. As suggested by the Cochrane Handbook (Higgins and Green, 2011), *I*^2^–values were interpreted with the following levels: 0–40, 30–60, 50–90, and 75–100%, meaning “Might not be important,” “Moderate,” “Substantial,” and “Considerable,” respectively. Heterogeneity, with a *p* < 0.1 considered significant.

(Egger's tests and funnel plots have not been carried out to assess any publication bias, because there was only a low amount of the studies included).

All analyses were performed by R environment (R Core Team, 2021).

To assess the certainty of the evidence we used the GRADE approach (Higgins and Green, 2011), which has four domains (risk of bias; inconsistency; indirectness; imprecision). The GRADE approach has four levels of evidence: high, moderate, low and very low. If there was a serious concern for any of the domains, we downgraded the evidence level.

## Results

### Systematic Search and Selection

**Figure 1:**
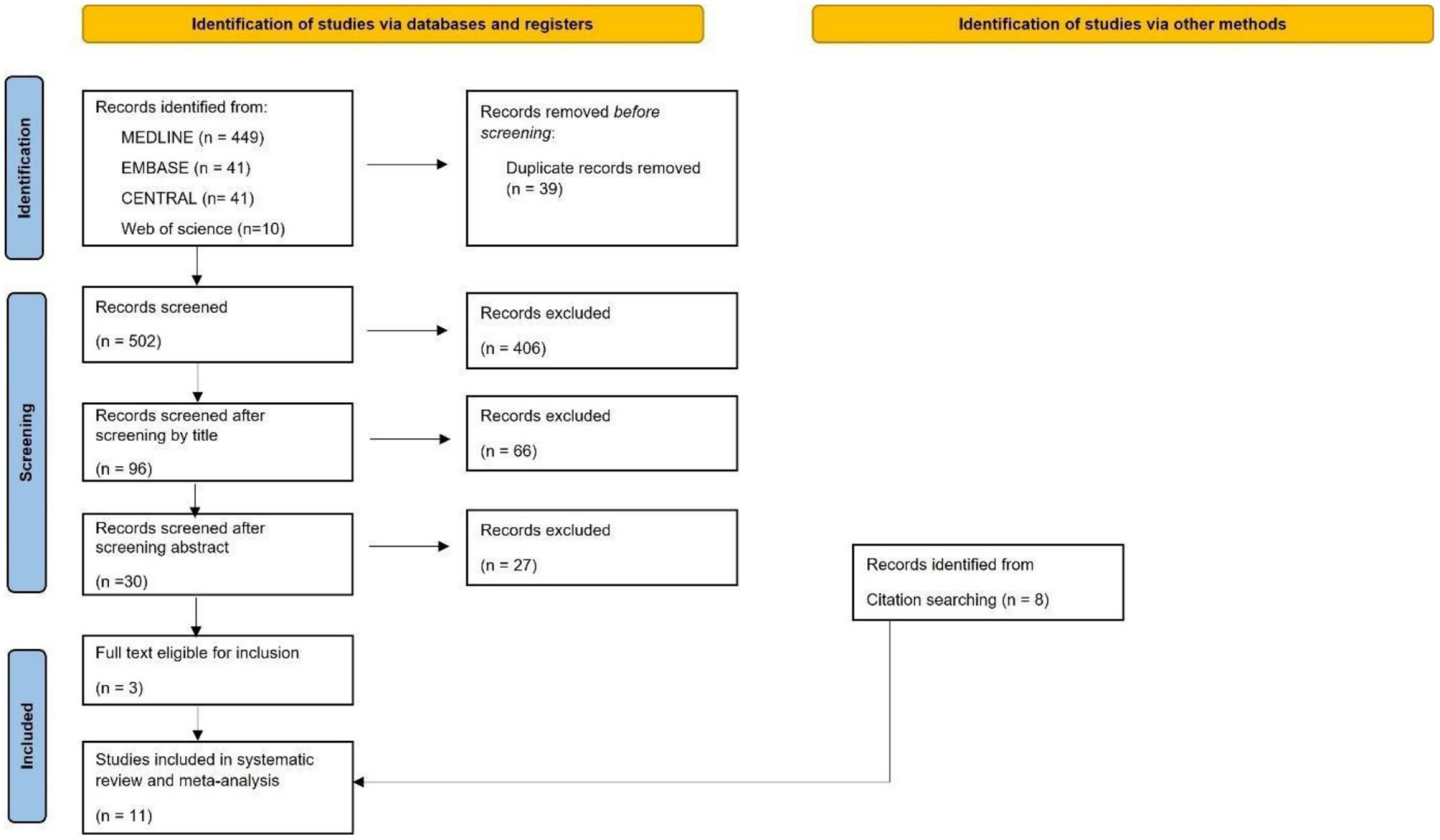
PRISMA flowchart for the study selection process (Page et al., 2021).

The systematic search yielded 541 records. After the automatic and manual removal of duplicates 502 records remained. The flowchart of the publication selection is presented in Figure 1. After checking the records and citation searching 11 publications remained. Five studies (Brunet et al., 2003; Eack et al., 2013; Roux et al., 2016a; Okruszek et al., 2018; Kronbichler et al., 2019) were included in the quantitative synthesis, and 9 studies (Das et al., 2012; Eack et al., 2013; Roux et al., 2014, 2015, 2016a,b; Okruszek et al., 2017; Kronbichler et al., 2019; Patel et al., 2020) in the qualitative synthesis.

All records found were used in either the meta-analysis or the systematic review.

The 5 studies (Brunet et al., 2003; Das et al., 2012; Eack et al., 2013; Roux et al., 2016a; Okruszek et al., 2017, 2018; Kronbichler et al., 2019) included in the quantitative synthesis examined 126 patients, while the 9 (Das et al., 2012; Eack et al., 2013; Roux et al., 2014, 2015, 2016a,b; Okruszek et al., 2017; Kronbichler et al., 2019; Patel et al., 2020) records included in the systematic review examined 157 patients (There is a complete overlap between patients in the studies by Roux et al. (2014, 2015, 2016a,b).

The characteristics of included studies are in Table 1 and Table 1 in the Supplementary Material.

**Table 1:** Characteristics of the included studies.

	**Task design**	**No. of patients (female of total %)**	**Age of patients (mean + SD)**	**No. of controls (female of total %)**	**Age of controls (mean + SD)**	**Medication (antipsychotic)**
Brunet et al. (2003)	PET CT during non-verbal task (select a correct ending)	7 (0.0)	31.0 ± 6.5	8 (0.0)	23.3 ± 1.68	All
Das et al. (2012)	fMRI during animated triangle task	20 (0.0)	34.5 ± 8.4	21 (0.0)	33.5 ± 8.4	All except one
Eack et al. (2013)	Perspective-Taking task	20 (30.0)	27.8 ± 6.61	20 (35.0)	26.5 ± 5.8	All except one
Kronbichler et al. (2019)	Perspective-Taking task	24 (0.0)	26.0 ± 5.1	24 (0.0)	25.7 ± 4.5	All
Okruszek et al. (2017)	Interpersonal detection task	25 (3.0)	35.7 ± 6.9	26 (48.0)	35.3 ± 7.1	All except one
Okruszek et al. (2018)	Interpersonal detection task	46 (32.6)	33.4 ± 7.0	40 (50.0)	30.2 ± 10.7	–
Patel et al. (2020)	Eye movement measurement during TASIT videos	39 (25.6)	40.6 ± 11.0	27 (40.7)	35.2 ± 9.3	All
Roux et al. (2014)	Eye movements measurements during animated cartoons	29 (27.6)	39.0 ± 12.5	29 (34.5)	40.7 ± 13.5	All
Roux et al. (2015)	Eye movements measurements during intentional motion perception	29 (27.6)	39.0 ± 12.5	29 (34.5)	40.7 ± 13.5	All
Roux et al. (2016a)	Eye movement measurement during non-verbal task (select the correct ending)	29 (27.6)	39.0 ± 12.5	29 (34.5)	40.7 ± 13.5	All
Roux et al. (2016b)	Eye movement measurement during Frith-Happé animation	29 (27.6)	39.0 ± 12.5	29 (34.5)	40.7 ± 13.5	All

### Accuracy

**Figure 2:**
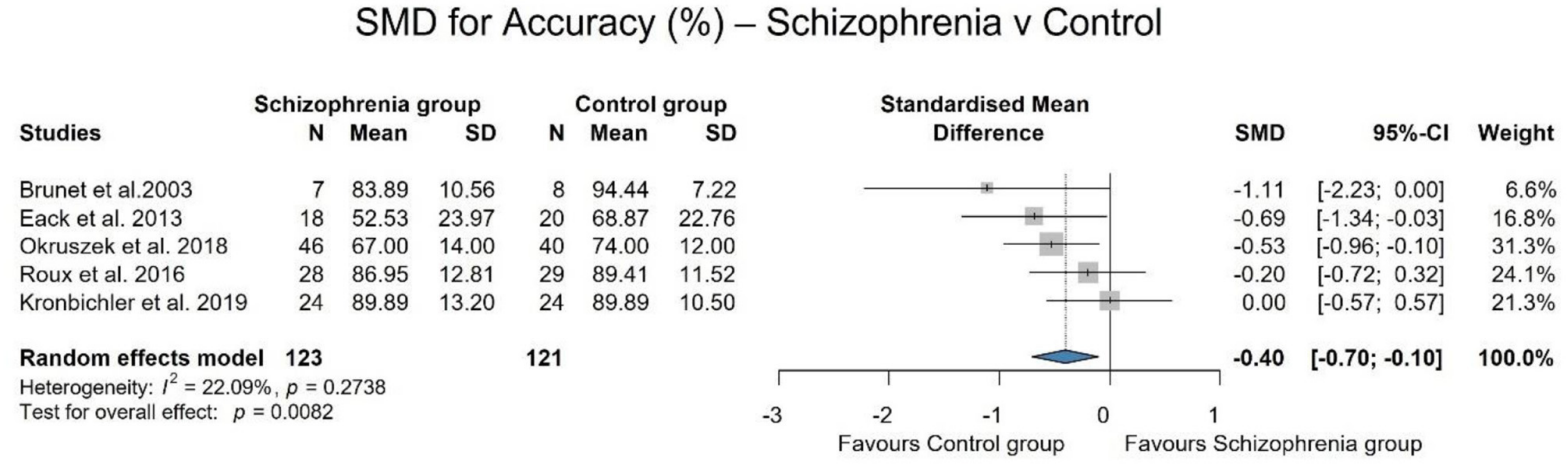
SMD for accuracy between patients with schizophrenia and control group. Patients with schizophrenia performed significantly poorer than controls.

For accuracy, data from 5 studies (Brunet et al., 2003; Eack et al., 2013; Roux et al., 2016a; Okruszek et al., 2018; Kronbichler et al., 2019) (which used different paradigms) were used, involving 123 patients and 121 controls. There is a significant difference [SMD = −0.40; 95% CI (−0.70, −0.10); *p* = 0.008] between patients with schizophrenia and controls with negligible statistical heterogeneity (*I*^2^ = 22.0%) in performance during implicit mentalizing tasks. On average, schizophrenic patients have a weaker performance with an effect size of −0.40, which is considered medium effect. The results are shown in Figure 2.

**Figure 3:**
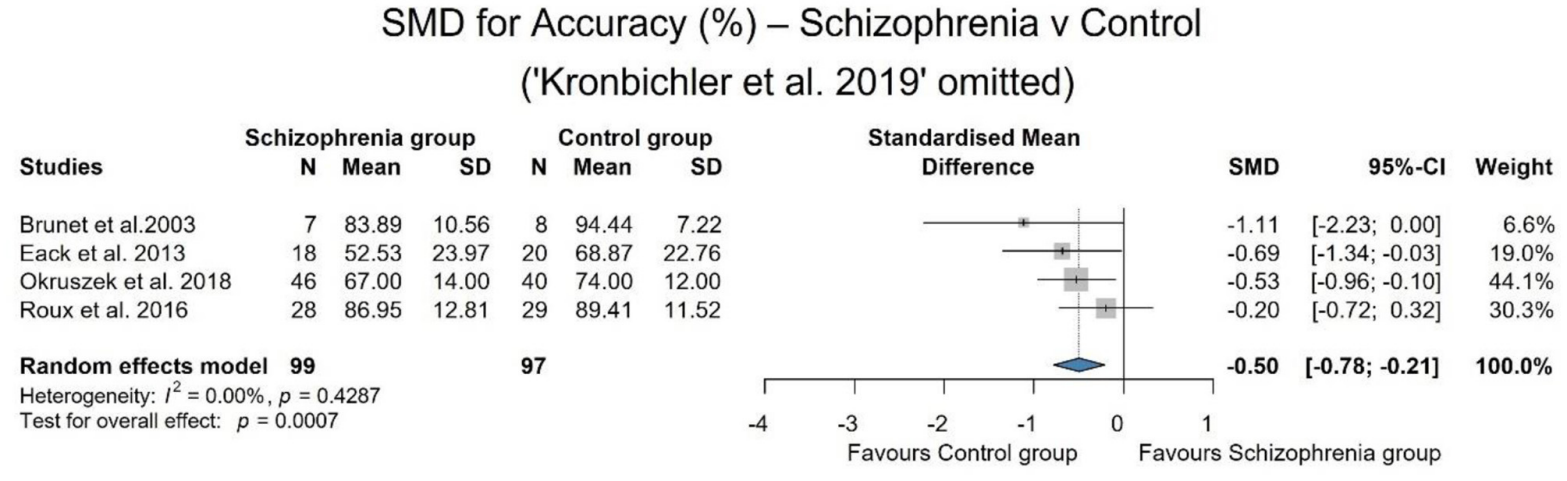
SMD for accuracy after leaving one out sensitivity analysis. It demonstrates that when heterogeneity decreases, the sample becomes homogeneous.

As the performance of the two groups was identical in one study (Kronbichler et al., 2019) and we considered that the question asked was significantly simpler than in the other studies, we conducted a leave one out sensitivity analysis during which the heterogeneity decreased, the studies become completely homogeneous (*I*^2^ = 0.0%) and the result remained significant and the effect size increased [SMD: −0.50; 95% CI (−0.78, −0.21); *p* = 0.001]. The results are summarized in Figure 3.

### Reaction Time

**Figure 4:**
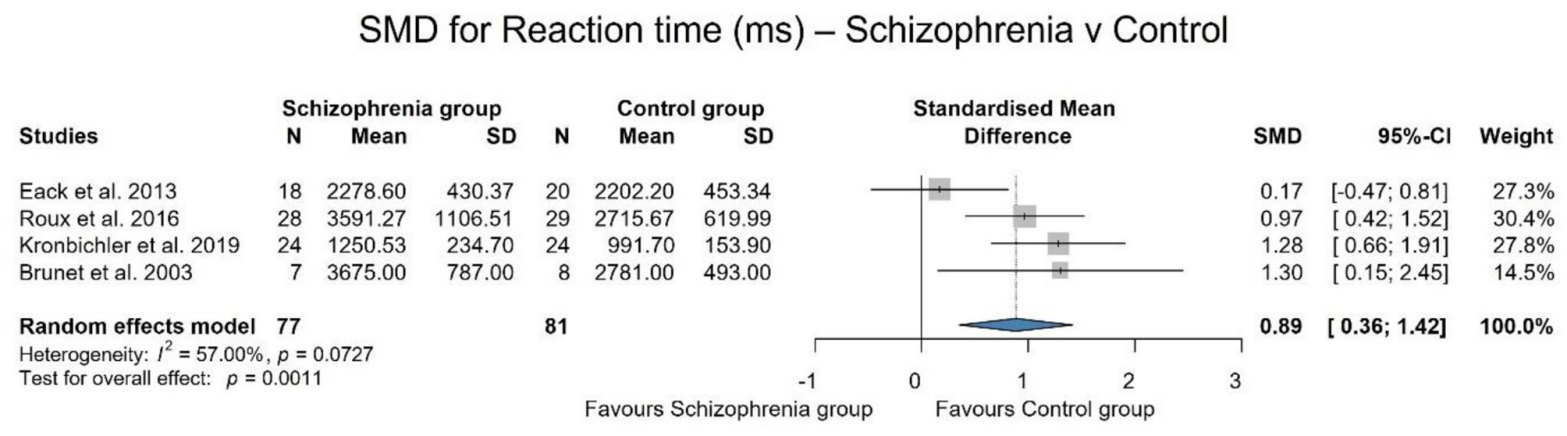
Forest plot representing that patients with schizophrenia have significantly longer reaction times.

For reaction time, data from 4 studies (Brunet et al., 2003; Eack et al., 2013; Roux et al., 2016a; Kronbichler et al., 2019) (using different paradigms) were used, including 77 patients and 81 controls. There is a significant difference in reaction time between the two groups [SMD: 0.89; 95% CI (0.36, 1.42); *p* = 0.001]. On average, the reaction time was significantly longer in the schizophrenic group compared to the control group with a large effect size (effect size: 0.89). There is a moderate heterogeneity (*I*^2^ = 57.00%). The forest plot showing the results can be found in Figure 4.

### GRADE Approach

The overall judgement of quality of evidence can be found in Table 2. The overall quality of evidence of the results was low/very low.

**Table 2:** GRADE approach.

**Outcome**	**No study/no patients/controls**	**Risk of bias**	**Inconsistency**	**Indirectness**	**Imprecision**	**Quality of evidence**
Accuracy	5/123/121	Not serious	Not serious	Not serious	Serious	Low
Reaction time	4/77/81	Not serious	Some	Not serious	Serious	Very low

### Systematic Review

#### FMRI Data

Concerning the comparison of activations/deactivations of schizophrenia patients and healthy subjects during mentalizing tasks, few data was available in the selected studies, which was not eligible for meta-analysis. Das et al. (2012) used an animated triangle task, consisting of mentalizing and control animations. In their study they emphasized a reduced activation in the right TPJ and both the right and left IFG in patients with schizophrenia compared to healthy controls during processing of mentalizing animation, and a similar activation in the left TPJ. The workgroup of Eack et al. (2013) employed a visual perspective-taking task, during which the schizophrenia group showed a reduced activation in the left OFC and in both the left and right anterior cingulate cortex (ACC), when compared to healthy individuals. During the same task, functional connectivity analyses were also conducted, which showed deficits in negative functional connectivity between the anterior cingulate and fusiform/parahippocampal gyrus in patients with schizophrenia. In controls the deactivation of the ACC was associated with an increase in activation in the right fusiform and parahippocampal gyri, while the schizophrenia group had the opposite correlation. In the study of Kronbichler et al. (2019) a different visual perspective-taking task was used. Concerning the activations a difference was found: the activations in the bilateral middle occipital gyrus (MOG; marginal group difference in left MOG) in the schizophrenia group were indifferent across task conditions, whereas controls showed an increased BOLD response in situations of spontaneous perspective-taking. Okruszek et al. (2017) used a setting in which the subjects had to decide whether two agents, presented by point-light displays were either communicating or acting independently. They emphasized the lower activation of the right posterior sulcus temporalis superior (STS) in patients with schizophrenia during communicative interactions as their main finding. In the functional connectivity analysis of the same task the control group showed an increased connectivity of the right posterior STS with structures associated with the mentalizing (bilateral STS and TPJ, mPFC). The patients on the other hand, activated structures linked with mentalizing network (left posterior STS/TPJ, right anterior STS), but to a lesser extent.

#### Eye Movements Measurements

Five studies used this measurement (Roux et al., 2014, 2015, 2016a,b; Patel et al., 2020) from whose data we could not make meta-analysis due to overlapping populations. They used different paradigms. Except for one study (Roux et al., 2016b), all described different eye movements in patients with schizophrenia compared to controls (Roux et al., 2014, 2015, 2016a; Patel et al., 2020). Three studies described reduced viewing time on the face (Roux et al., 2014, 2016a; Patel et al., 2020), one described that patients spent more time observing contextual cues, which also highlighted the importance of time, based on which patients' mentalizing is delayed (Roux et al., 2016a).

#### Fixation Duration

Three (Roux et al., 2014, 2016b; Patel et al., 2020) studies measured the mean fixation duration which refers to the processing of information. Two studies found increased mean fixation duration in patients, which may indicate less saccades, shorter scanning paths (Roux et al., 2014, 2016b). In addition, Roux et al. (2016b) demonstrated that in patients and in controls the length of the fixation increases along with the complexity of the task, which referees an equal increase in cognitive processing in both groups. In contrast, Patel et al. described more saccades, shorter fixation duration time in patients with schizophrenia (Patel et al., 2020).

#### Looking at Cues of Mentalization

Three of the 5 studies found that patients spent less time looking at the cues of mentalization (Roux et al., 2014, 2016a; Patel et al., 2020). One study described that patients looked at intentional cues as long as controls while watching Firth—Happé animations (animated triangles interactions) (Roux et al., 2016b). One study demonstrated that patients have more center looking strategy during intentional motion perception (used chasing detection paradigm) (Roux et al., 2015). Patel et al. showed that patients' eye position was more variable than controls; the average area of the eye position was larger in patients during watching TASIT videos (Patel et al., 2020). Patients spent less time viewing action regions of interest during intentional attribution tasks based on comic strips (Roux et al., 2016a). On the other hand, the looking time of the contextual regions was greater in patients than in controls especially when the processing of social context required attribution of intention (Roux et al., 2016a). Roux et al. also demonstrated that looking time of a displaced object was the same in both groups when participants watched animated cartoons, but patients with schizophrenia spent less time looking at the head of the agent, and the decreased sensitivity to goal and belief attribution was associated with decreased attention to gaze orientation (Roux et al., 2014).

#### Face Looking Time

Three studies measured the face looking time of the participants. All of them found that patients spent less time looking at facial expressions (Roux et al., 2014, 2016a; Patel et al., 2020). Two of them (Roux et al., 2014, 2016a) examined the central field of view, the third one (Patel et al., 2020) described this discrepancy in peripheral field of view.

### Risk of Bias Assessment

The overall risk of bias was low to high in the studies included. Detailed results of the quality assessment are found in Supplementary Materials.

## Discussion

Our meta-analysis and systematic review show that patients with schizophrenia have subtle impairments in implicit mentalizing. The results of the meta-analysis revealed more inaccurate performance, slower reaction times. The systematic review part of the analysis indicated different brain activation patterns; and different visual processing compared to control subjects during implicit mentalization tasks.

The patients with schizophrenia exhibited a small but significant impairment in accuracy with negligible statistical heterogeneity and a medium effect size. Decreasing heterogeneity during leave one out analysis may result from differences between the paradigms used in the studies, which may indicate the importance of the mentalizing tasks. The results suggest a subtle deficit compared to explicit mentalizing, which was found to be highly significantly impaired (Sprong et al., 2007; Bora et al., 2009). It is important to point out that the accuracy probably may not directly reflect the implicit mentalization, since it is measured indirectly and appropriateness of present paradigms for detecting implicit mentalizing is still under debate (e.g., Heyes, 2014; Santiesteban et al., 2014; Phillips et al., 2015; Kulke et al., 2019).

In contrast to accuracy, reaction time may reflect implicit mentalizing more closely (Kovács et al., 2010; Edwards and Low, 2017). Our meta-analysis revealed a significantly slower reaction time among patients with schizophrenia with a large effect size. It also suggests that implicit mentalizing is affected in patients with schizophrenia. However, it may also mean that a less efficient processing speed is responsible for the lower performance in implicit mentalizing tasks. Some of the studies found intact mentalizing but delay in intention attribution due to a slowdown in context processing (Roux et al., 2016a,b). It is also essential to highlight that moderate heterogeneity was found, which may result from the markedly different paradigms, and the differences in the difficulties of the tasks. For this outcome, the effect size is high, which highlights its practical significance, but the certainty for outcome assessments is very low. It is important to note that two of the four studies were perspective taking studies. Some research assumed that perspective-taking tasks do not examine implicit mentalizing but are determined by domain-general processes (Santiesteban et al., 2014; Cole et al., 2016), whereas other studies confirmed that these tasks are driven by implicit mentalization (Samson et al., 2010; Ferguson et al., 2015; Gardner et al., 2018).

Concerning functional imaging, differences in activation patterns during implicit mentalization tasks were found. Patients with schizophrenia recruited several temporal regions (e.g., STG, TPJ, STS), however they showed lower activity in these areas compared to controls (Das et al., 2012; Okruszek et al., 2017). It may indicate a less effective processing of social situations. These regions have an important role in detecting intentionality from biological salient cues (Sugranyes et al., 2011; Frith and Frith, 2012; Ciaramidaro et al., 2014), and they were found to be active during implicit and during explicit mentalizing (Molenberghs et al., 2016). One study (Das et al., 2012) found reduced activation in the right TPJ, but not in the left TPJ in patients with schizophrenia. TPJ is active both in implicit and explicit processing (Molenberghs et al., 2016). The observed pattern (Das et al., 2012) is the opposite that has been found recently in healthy people, when stronger activation was detected in the right TPJ compared to the activity on the left side (Boccadoro et al., 2019). However, the activation of temporal regions and TPJ in patients with schizophrenia may point to the process of appreciating the communicative nature of the interaction being relatively unaffected, although it may rely on a slightly different brain network activity. On the other hand, controls activated several occipital and occipitotemporal regions which may suggest a less efficient computation of spontaneous visual perspective taking (Kronbichler et al., 2019). Some further common areas of explicit and implicit processing (Molenberghs et al., 2016), such as bilateral IFG (Das et al., 2012) and the cingulate gyrus (Eack et al., 2013) was found under-activated in subjects with schizophrenia relative to healthy controls. Furthermore, reduced activation of the OFC, which is thought to be an important part of the implicit mentalizing network (Molenberghs et al., 2016), was also reported in patients compared to controls (Eack et al., 2013). Overall, these results suggest that patients with schizophrenia are likely to detect communicative intentions, but they may activate a different, and possibly less integrated neural network during implicit mentalizing. As the connectivity analyses (Eack et al., 2013; Okruszek et al., 2017) revealed there are important differences in network connectivity in patients with schizophrenia. The decreased connectivity of the concerned areas (posterior STS, medial pre-frontal, and medial-temporal regions) possibly has a role in the less efficient implicit mentalizing. It may correspond to recent reports that found an alteration in the integrity of the neuronal network that is responsible for the processing of low-level pre-reflective intention detection, and also a diminished between-network connectivity of the low-level (implicit) and higher-level (explicit) mentalizing networks (Choe et al., 2018).

The qualitative synthesis revealed some further characteristics of implicit mentalizing in schizophrenia. Patients with schizophrenia showed subtle deficits in visual processing, which is not surprising since studies of implicit mentalizing have predominantly used visual paradigms. It seems a relatively consistent result that visual scanning is inadequate and slower in patients than in controls (Roux et al., 2015; Patel et al., 2020). Patients tend to focus more on contextual cues instead of processing socially relevant cues (Roux et al., 2014). In addition, patients fixate less on the head region (Roux et al., 2014, 2016a), especially when the face is in the peripheral field of view. They tend to bring faces less frequently into the central field of view for processing facial expressions (Patel et al., 2020). According to these results, patients with schizophrenia seem to focus less on socially relevant cues, especially on human faces, although face processing has a central role in detecting mental states (Itier and Batty, 2009). It is also important that interfering stimuli can significantly impair processing (e.g., stimulus overload) (Roux et al., 2016b). These results on visual processing and mentalizing seem to correspond with a recent meta-analysis, which found that mentalizing is linked to several neurocognitive deficits in schizophrenia, and that the type of the task used is a significant moderator in these relationships (Thibaudeau et al., 2020). This in turn also suggests that the complexity of the social situation and the deficient neurocognitive abilities may limit the efficiency of implicit mentalizing.

Our systematic review and meta-analysis have several limitations. The main limitation is the low number of eligible studies. However, we should mention that according to the Cochrane Handbook for Systematic Reviews a meta-analysis is the statistical combination of results from two or more separate studies (Higgins and Green, 2011). In addition, as claimed by Valentine et al. at least two studies are sufficient for a meta-analysis, because it is still the most transparent and valid way of synthesizing research data (Valentine et al., 2010). Based on this approach, we thought that the significance of the topic deserves a summary of the available data. We also found 8 studies via other methods than systematic search (e.g., checking the references of the selected articles), which is a significant limitation. Several search keys were tried, but the number of eligible records found did not differ significantly. This was probably due to the lack of or inconsistent use of terms “implicit,” “explicit,” or “spontaneous” in publications that may have distorted the selection results. It is also important to mention that several different terms are used for mentalizing as well (Theory of Mind, mentalization, perspective taking, intentionality, mindreading, etc.) Unfortunately, a few studies were excluded (and included in the systematic part) because they did not provide sufficient data for meta-analysis. We contacted the authors, but not all missing data were received. Another limitation is that some studies have examined schizoaffective patients in addition to schizophrenic patients, which may also raise the bias. However, schizoaffective disorder is regularly included in schizophrenia studies as a schizophrenia-spectrum disorder.

It is important to highlight that other factors could also influence the results, but unfortunately, due to the small number of studies, we could not perform meta-regression [which requires at least 10 studies (Higgins and Green, 2011)], to assess the effect of moderator variables (e.g., symptom severity, general intelligence, age, education, gender ratio, duration of illness, etc.).

Another important limitation is the moderate heterogeneity due to different methods (different stimuli were used, different questions were asked) used to measure implicit mentalizing. Further limitations are that the studies involved have a small number of cases and most of the patients were taking medication at the time of the studies.

In conclusion, our results do not allow a firm conclusion at this moment. The substantial heterogeneity of the paradigms used in a small number of studies limit the generalizability of the results. Overall, in addition to the deficit of explicit mentalizing, implicit mentalizing performance is also affected in schizophrenia, if not to the same extent. It seems likely that some elements of implicit mentalizing might be relatively unaffected (e.g., detection of intentionality (Roux et al., 2016b; Okruszek et al., 2018), but the effectiveness may be limited by certain neurocognitive deficits. However, it would be important to have a clearer picture of the nature of implicit mentalizing in schizophrenia because it may significantly affect the remediation of mentalizing skills (Langdon et al., 2017), which in turn highlights the necessity of further studies.

## Data Availability Statement

The original contributions presented in the study are included in the article/Supplementary Material, further inquiries can be directed to the corresponding author/s.

## Author Contributions

TC and AH: data collection, data analysis, writing article, and manuscript revision. SK, FD, MV-S, and ZS: data analysis and manuscript revision. MK, MH, and EV: data collection. PH: study design. TT: study design and manuscript revision. RH: study idea, study design, writing article, and manuscript revision. All authors contributed to the article and approved the submitted version.

## Funding

This work was supported by National Brain Research Program Grant No. NAP KTIA NAP-A-II/12(2017-2021) and the National Excellence programme (2019-2021, FIKP II).

## Conflict of Interest

The authors declare that the research was conducted in the absence of any commercial or financial relationships that could be construed as a potential conflict of interest.

## Publisher's Note

All claims expressed in this article are solely those of the authors and do not necessarily represent those of their affiliated organizations, or those of the publisher, the editors and the reviewers. Any product that may be evaluated in this article, or claim that may be made by its manufacturer, is not guaranteed or endorsed by the publisher.

## References

[B1] Alexander-Bloch A. F. Reiss P. T. Rapoport J. McAdams H. Giedd J. N. Bullmore E. T. . (2014). Abnormal cortical growth in schizophrenia targets normative modules of synchronized development. Biol. Psychiatry 76, 438–446. 10.1016/j.biopsych.2014.02.010 24690112 PMC4395469

[B2] Apperly I. A. Butterfill S. A. (2009). Do humans have two systems to track beliefs and belief-like states? Psychol. Rev. 116, 953–970. 10.1037/a0016923 19839692

[B3] Baillargeon R. Scott R. M. He Z. Sloane S. Setoh P. Jin K.-S. . (2015). Psychological and sociomoral reasoning in infancy, in APA Handbook of Personality and Social Psychology, Vol. 1, eds Borgida E. Bargh J. A. (Washington, DC: American Psychological Association), 79–150. 10.1037/14341-003

[B4] Benedetti F. Bernasconi A. Bosia M. Cavallaro R. Dallaspezia S. Falini A. . (2009). Functional and structural brain correlates of theory of mind and empathy deficits in schizophrenia. Schizophr. Res. 114, 154–160. 10.1016/j.schres.2009.06.021 19632816

[B5] Boccadoro S. Cracco E. Hudson A. R. Bardi L. Nijhof A. D. Wiersema J. R. . (2019). Defining the neural correlates of spontaneous theory of mind (ToM): an fMRI multi-study investigation. Neuroimage 203, 116–193 10.1016/j.neuroimage.2019.116193 31525499

[B6] Bora E. Pantelis C. (2013). Theory of mind impairments in first-episode psychosis, individuals at ultra-high risk for psychosis and in first-degree relatives of schizophrenia: systematic review and meta-analysis. Schizophr. Res. 144, 31–36. 10.1016/j.schres.2012.12.013 23347949

[B7] Bora E. Yucel M. Pantelis C. (2009). Theory of mind impairment in schizophrenia: meta-analysis. Schizophr. Res. 109, 1–9. 10.1016/j.schres.2008.12.020 19195844

[B8] Brunet E. Sarfati Y. Hardy-Bayle M. C. Decety J. (2003). Abnormalities of brain function during a nonverbal theory of mind task in schizophrenia. Neuropsychologia 41, 1574–1582. 10.1016/S0028-3932(03)00119-2 12887982

[B9] Buttelmann D. Carpenter M. Tomasello M. (2009). Eighteen-month-old infants show false belief understanding in an active helping paradigm. Cognition 112, 337–342. 10.1016/j.cognition.2009.05.006 19524885

[B10] Butterfill S. A. Apperly I. A. (2013). How to construct a minimal theory of mind. Mind Lang. 28, 606–637. 10.1111/mila.12036

[B11] Choe E. Lee T. Y. Kim M. Hur J. W. Yoon Y. B. Cho K. K. . (2018). Aberrant within- and between-network connectivity of the mirror neuron system network and the mentalizing network in first episode psychosis. Schizophr. Res. 199, 243–249. 10.1016/j.schres.2018.03.024 29599093

[B12] Ciaramidaro A. Becchio C. Colle L. Bara B. G. Walter H. (2014). Do you mean me? Communicative intentions recruit the mirror and the mentalizing system. Soc. Cogn. Affect. Neurosci. 9, 909–916. 10.1093/scan/nst062 23620602 PMC4090952

[B13] Cole G. G. Atkinson M. Le A. T. Smith D. T. (2016). Do humans spontaneously take the perspective of others? Acta Psychol. 164, 165–168. 10.1016/j.actpsy.2016.01.007 26826864

[B14] Das P. Lagopoulos J. Coulston C. M. Henderson A. F. Malhi G. S. (2012). Mentalizing impairment in schizophrenia: a functional MRI study. Schizophr. Res. 134, 158–164. 10.1016/j.schres.2011.08.019 21943555

[B15] Davidson C. A. Piskulic D. Addington J. Cadenhead K. S. Cannon T. D. Cornblatt B. A. . (2018). Age-related trajectories of social cognition in youth at clinical high risk for psychosis: an exploratory study. Schizophr. Res. 201, 130–136. 10.1016/j.schres.2018.05.001 29751984 PMC8130825

[B16] DerSimonian R. Laird N. (1986). Meta-analysis in clinical trials. Control. Clin. Trials 7, 177–188. 10.1016/0197-2456(86)90046-2 3802833

[B17] Eack S. M. Wojtalik J. A. Newhill C. E. Keshavan M. S. Phillips M. L. (2013). Prefrontal cortical dysfunction during visual perspective-taking in schizophrenia. Schizophr. Res. 150, 491–497. 10.1016/j.schres.2013.08.022 24055199 PMC3825745

[B18] Edwards K. Low J. (2017). Reaction time profiles of adults' action prediction reveal two mindreading systems. Cognition 160, 1–16. 10.1016/j.cognition.2016.12.004 28024170

[B19] El Kaddouri R. Bardi L. De Bremaeker D. Brass M. Wiersema J. R. (2020). Measuring spontaneous mentalizing with a ball detection task: putting the attention-check hypothesis by Phillips and colleagues (2015) to the test. Psychol. Res. 84, 1749–1757. 10.1007/s00426-019-01181-7 30976921

[B20] Ferguson H. J. Apperly I. Ahmad J. Bindemann M. Cane J. (2015). Task constraints distinguish perspective inferences from perspective use during discourse interpretation in a false belief task. Cognition 139, 50–70. 10.1016/j.cognition.2015.02.010 25800351

[B21] Frith C. D. Frith U. (2012). Mechanisms of social cognition. Annu. Rev. Psychol. 63, 287–313. 10.1146/annurev-psych-120710-100449 21838544

[B22] Gardner M. R. Bileviciute A. P. Edmonds C. J. (2018). Implicit mentalising during level-1 visual perspective-taking indicated by dissociation with attention orienting. Vision 2, 3. 10.3390/vision2010003 31735867 PMC6836282

[B23] Gogtay N. (2008). Cortical brain development in schizophrenia: insights from neuroimaging studies in childhood-onset schizophrenia. Schizophr. Bull. 34, 30–36. 10.1093/schbul/sbm103 17906336 PMC2632387

[B24] Grosse Wiesmann C. Friederici A. D. Singer T. Steinbeis N. (2020). Two systems for thinking about others' thoughts in the developing brain. Proc. Natl. Acad. Sci. U.S.A. 117, 6928–6935. 10.1073/pnas.1916725117 32152111 PMC7104351

[B25] Hayden J. A. van der Windt D. A. Cartwright J. L. Côté P. Bombardier C. (2013). Assessing bias in studies of prognostic factors. Ann. Intern. Med. 158, 280–286. 10.7326/0003-4819-158-4-201302190-00009 23420236

[B26] Healey K. M. Penn D. L. Perkins D. Woods S. W. Addington J. (2013). Theory of mind and social judgments in people at clinical high risk of psychosis. Schizophr. Res. 150, 498–504. 10.1016/j.schres.2013.08.038 24055202 PMC3971540

[B27] Herold R. Feldmann A. Simon M. Tényi T. Kövér F. Nagy F. . (2009). Regional gray matter reduction and theory of mind deficit in the early phase of schizophrenia: a voxel-based morphometric study. Acta Psychiatr. Scand. 119, 199–208. 10.1111/j.1600-0447.2008.01297.x 19016669

[B28] Herold R. Tényi T. Lénárd K. Trixler M. (2002). Theory of mind deficit in people with schizophrenia during remission. Psychol. Med. 32, 1125–1129. 10.1017/S0033291702005433 12214792

[B29] Herold R. Varga E. Hajnal A. Hamvas E. Berecz H. Tóth B. . (2018). Altered neural activity during irony comprehension in unaffected first-degree relatives of schizophrenia patients-an fMRI study. Front. Psychol. 8:2309. 10.3389/fpsyg.2017.02309 29375430 PMC5767266

[B30] Heyes C. (2014). Submentalizing: I am not really reading your mind. Perspect. Psychol. Sci. 9, 131–143. 10.1177/1745691613518076 26173251

[B31] Higgins J. P. T. Green S (Eds.). (2011). Cochrane Handbook for Systematic Reviews of Interventions. Version 5.1.0 (Updated March 2011). The Cochrane Collaboration.

[B32] Horan W. P. Nuechterlein K. H. Wynn J. K. Lee J. Castelli F. Green M. F. (2009). Disturbances in the spontaneous attribution of social meaning in schizophrenia. Psychol. Med. 39, 635–643. 10.1017/S0033291708003838 18606048 PMC2903627

[B33] Hyde D. C. Simon C. E. Ting F. Nikolaeva J. I. (2018). Functional organization of the temporal-parietal junction for theory of mind in preverbal infants: a near-infrared spectroscopy study. J. Neurosci. 38, 4264–4274. 10.1523/JNEUROSCI.0264-17.2018 29593053 PMC6596006

[B34] Itier R. J. Batty M. (2009). Neural bases of eye and gaze processing: the core of social cognition. Neurosci. Biobehav. Rev. 33, 843–863. 10.1016/j.neubiorev.2009.02.004 19428496 PMC3925117

[B35] Kampis D. Parise E. Csibra G. Kovács Á. M. (2015). Neural signatures for sustaining object representations attributed to others in preverbal human infants. Proc. R. Soc. B 282:20151683. 10.1098/rspb.2015.1683 26559949 PMC4685805

[B36] Koelkebeck K. Hirao K. Miyata J. Kawada R. Saze T. Dannlowski U. . (2013). Impact of gray matter reductions on theory of mind abilities in patients with schizophrenia. Soc. Neurosci. 8, 631–639. 10.1080/17470919.2013.837094 24047258

[B37] Kovács Á. M. Téglás E. Endress A. D. (2010). The social sense: susceptibility to others' beliefs in human infants and adults. Science 330, 1830–1834. 10.1126/science.1190792 21205671

[B38] Kronbichler L. Stelzig-schöler R. Pearce B. G. Tschernegg M. Said-Yürekli S. Crone J. S. . (2019). Reduced spontaneous perspective taking in schizophrenia. Psychiatry Res. Neuroimaging. 292:5–12. 10.1016/j.pscychresns.2019.08.007 31472416

[B39] Kronbichler L. Tschernegg M. Martin A. I. Schurz M. Kronbichler M. (2017). Abnormal brain activation during theory of mind tasks in schizophrenia: a meta-analysis. Schizophr. Bull. 43, 1240–1250. 10.1093/schbul/sbx073 28575475 PMC5737081

[B40] Kulke L. Hinrichs M. A. B. (2021). Implicit theory of mind under realistic social circumstances measured with mobile eye-tracking. Sci. Rep. 11:1215. 10.1038/s41598-020-80614-5 33441890 PMC7806733

[B41] Kulke L. Wübker M. Rakoczy H. (2019). Is implicit theory of mind real but hard to detect? Testing adults with different stimulus materials. R. Soc. Open Sci. 6:190068. 10.1098/rsos.190068 31417713 PMC6689622

[B42] Langdon R. Flynn M. Connaughton E. Brüne M. (2017). Impairments of spontaneous and deliberative mentalizing co-occur, yet dissociate, in schizophrenia. Br. J. Clin. Psychol. 56, 372–387. 10.1111/bjc.12144 28603875

[B43] Lieberman M. D. (2007). Social cognitive neuroscience: a review of core processes. Ann. Rev. Psychol. 58, 259–289. 10.1146/annurev.psych.58.110405.085654 17002553

[B44] Low J. Watts J. (2013). Attributing false beliefs about object identity reveals a signature blind spot in humans' efficient mind-reading system. Psychol. Sci. 24, 305–311. 10.1177/0956797612451469 23307943

[B45] Marjoram D. Job D. E. Whalley H. C. Gountouna V. E. McIntosh A. M. Simonotto E. . (2006). A visual joke fMRI investigation into theory of mind and enhanced risk of schizophrenia. Neuroimage 31, 1850–1858. 10.1016/j.neuroimage.2006.02.011 16624578

[B46] Martin A. K. Robinson G. Dzafic I. Reutens D. Mowry B. (2014). Theory of mind and the social brain: implications for understanding the genetic basis of schizophrenia. Genes Brain Behav. 13, 104–117. 10.1111/gbb.12066 23927712

[B47] Molenberghs P. Johnson H. Henry J. D. Mattingley J. B. (2016). Understanding the minds of others: a neuroimaging meta-analysis. Neurosci. Biobehav. Rev. 65:276–291. 10.1016/j.neubiorev.2016.03.020 27073047

[B48] Nijhof A. D. Brass M. Bardi L. Wiersema J. R. (2016). Measuring mentalizing ability: a within-subject comparison between an explicit and implicit version of a ball detection task. PLoS ONE 11:e0164373. 10.1371/journal.pone.0164373 27723814 PMC5056736

[B49] Okruszek Ł. Piejka A. Wysokiński A. Szczepocka E. Manera V. (2018). Biological motion sensitivity, but not interpersonal predictive coding is impaired in schizophrenia. J. Abnorm. Psychol. 127, 305–313. 10.1037/abn0000335 29369645

[B50] Okruszek L. Wordecha M. Jarkiewicz M. Kossowski B. Lee J. Marchewka A. (2017). Brain correlates of recognition of communicative interactions from biological motion in schizophrenia. Psychol. Med. 48, 1862–1871. 10.1017/S0033291717003385 29173243

[B51] Onishi K. H. Baillargeon R. (2005). Do 15-month-old infants understand false beliefs? Science 308, 255–258. 10.1126/science.1107621 15821091 PMC3357322

[B52] Page M. J. McKenzie J. E. Bossuyt P. M. Boutron I. Hoffmann T. C. Mulrow C. D. . (2021). The PRISMA 2020 statement: an updated guideline for reporting systematic reviews. BMJ 372, n71. 10.1136/bmj.n71 33782057 PMC8005924

[B53] Patel G. H. Arkin S. C. Ruiz-Betancourt D. R. DeBaun H. M. Strauss N. E. Bartel L. P. . (2020). What you see is what you get: visual scanning failures of naturalistic social scenes in schizophrenia. Psychol. Med. 10.1017/S0033291720001646. [Epub ahead of print]. 32498743 PMC7751380

[B54] Phillips J. Ong D. C. Surtees A. D. Xin Y. Williams S. Saxe R. . (2015). A second look at automatic theory of mind: reconsidering Kovács, Téglás, and Endress (2010). Psychol. Sci. 26, 1353–1367. 10.1177/0956797614558717 26253550

[B55] R Core Team (2021). R: A Language and Environment for Statistical Computing. R version 4.1.2 (2021-11-01). Vienna: R Foundation for Statistical Computing.

[B56] Roux P. Brunet-Gouet E. Passerieux C. Ramus F. (2016a). Eye-tracking reveals a slowdown of social context processing during intention attribution in patients with schizophrenia. J. Psychiatry Neurosci. 41, E13–E21. 10.1503/jpn.150045 26836621 PMC4764486

[B57] Roux P. Forgeot D'arc B. Passerieux C. Ramus F. (2014). Is the theory of mind deficit observed in visual paradigms in schizophrenia explained by an impaired attention toward gaze orientation? Schizophr. Res. 157, 78–83. 10.1016/j.schres.2014.04.031 24857238

[B58] Roux P. Passerieux C. Ramus F. (2015). An eye-tracking investigation of intentional motion perception in patients with schizophrenia. J. Psychiatry Neurosci. 40, 118–125. 10.1503/jpn.140065 25247443 PMC4354817

[B59] Roux P. Smith P. Passerieux C. Ramus F. (2016b). Preserved implicit mentalizing in schizophrenia despite poor explicit performance: evidence from eye tracking. Sci. Rep. 6, 34728. 10.1038/srep34728 27703225 PMC5050453

[B60] Samson D. Apperly I. A. Braithwaite J. Andrews B. (2010). Seeing it your way: evidence for altercentric intrusion effects in visual perspective taking. J. Exp. Psychol. Hum. Percept. Perform. 36, 1255–1266. 10.1037/a0018729 20731512

[B61] Santiesteban I. Catmur C. Hopkins S. C. Bird G. Heyes C. (2014). Avatars and arrows: implicit mentalizing or domain-general processing? J. Exp. Psychol. Hum. Percept. Perform. 40, 929–937. 10.1037/a0035175 24377486

[B62] Schneider D. Bayliss A. P. Becker S. I. Dux P. E. (2012). Eye movements reveal sustained implicit processing of others' mental states. J. Exp. Psychol. Gen. 141, 433–438. 10.1037/a0025458 21910557

[B63] Scott R. M. Baillargeon R. (2017). Early false-belief understanding. Trends Cogn. Sci. 21, 237–249. 10.1016/j.tics.2017.01.012 28259555

[B64] Selemon L. D. Zecevic N. (2015). Schizophrenia: a tale of two critical periods for prefrontal cortical development. Transl. Psychiatry 8, e623. 10.1038/tp.2015.115 26285133 PMC4564568

[B65] Senju A. (2012). Spontaneous theory of mind and its absence in autism spectrum disorders. Neuroscientist 18, 108–113. 10.1177/1073858410397208 21609942 PMC3796729

[B66] Senju A. (2013). Atypical development of spontaneous social cognition in autism spectrum disorders. Brain Dev. 35, 96–101. 10.1016/j.braindev.2012.08.002 22964276

[B67] Southgate V. Chevallier C. Csibra G. (2010). Seventeen-month-olds appeal to false beliefs to interpret others' referential communication. Dev. Sci. 13, 907–912. 10.1111/j.1467-7687.2009.00946.x 20977561

[B68] Southgate V. Senju A. Csibra G. (2007). Action anticipation through attribution of false belief by 2-year-olds. Psychol. Sci. 18, 587–592. 10.1111/j.1467-9280.2007.01944.x 17614866

[B69] Sprong M. Schothorst P. Vos E. Hox J. van Engeland H. (2007). Theory of mind in schizophrenia: meta-analysis. Br. J. Psychiatry. 191:5–13. 10.1192/bjp.bp.107.035899 17602119

[B70] Sugranyes G. Kyriakopoulos M. Corrigall R. Taylor E. Frangou S. (2011). Autism spectrum disorders and schizophrenia: meta-analysis of the neural correlates of social cognition. PLoS ONE 6, e25322. 10.1371/journal.pone.0025322 21998649 PMC3187762

[B71] Thibaudeau É. Achim A. M. Parent C. Turcotte M. Cellard C. (2020). A meta-analysis of the associations between theory of mind and neurocognition in schizophrenia. Schizophr. Res. 216, 118–128. 10.1016/j.schres.2019.12.017 31899095

[B72] Tikka D. L. Singh A. R. Tikka S. K. (2020). Social cognitive endophenotypes in schizophrenia: a study comparing first episode schizophrenia patients and, individuals at clinical- and familial- 'at-risk' for psychosis. Schizophr. Res. 215, 157–166. 10.1016/j.schres.2019.10.053 31761472

[B73] Valentine J. C. Pigott T. D. Rothstein H. R. (2010). How many studies do you need? A primer on statistical power for meta-analysis. J. Educ. Behav. Stat. 2, 215–247. 10.3102/1076998609346961

[B74] van der Wel R. P. Sebanz N. Knoblich G. (2014). Do people automatically track others' beliefs? Evidence from a continuous measure. Cognition 130, 128–133. 10.1016/j.cognition.2013.10.004 24216021

[B75] Van Overwalle F. Vandekerckhove M. (2013). Implicit and explicit social mentalizing: dual processes driven by a shared neural network. Front. Hum. Neurosci. 7, 560. 10.3389/fnhum.2013.00560 24062663 PMC3772308

[B76] Velthorst E. Fett A. J. Reichenberg A. Perlman G. van Os J. Bromet E. J. . (2017). The 20-year longitudinal trajectories of social functioning in individuals with psychotic disorders. Am. J. Psychiatry 174, 1075–1085. 10.1176/appi.ajp.2016.15111419 27978770 PMC5474222

[B77] Vogeley K. (2017). Two social brains: neural mechanisms of intersubjectivity. Philos. Trans. R. Soc. Lond. B Biol. Sci. 372, 20160245. 10.1098/rstb.2016.0245 28673921 PMC5498305

[B78] Vucurovic K. Caillies S. Kaladjian A. (2021). Neural correlates of mentalizing in individuals with clinical high risk for schizophrenia: ALE meta-analysis. Front Psychiatry 12:634015. 10.3389/fpsyt.2021.634015 33959048 PMC8095711

[B79] Weinberger D. R. (1987). Implications of normal brain development for the pathogenesis of schizophrenia. Arch. Gen. Psychiatry 44, 660–669. 10.1001/archpsyc.1987.01800190080012 3606332

