# Supplementary material for: Implicit Mentalizing in Patients With Schizophrenia: A Systematic Review and Meta-Analysis
Source: Front Psychol. 2022 Feb 2;13:790494. doi: 10.3389/fpsyg.2022.790494 (PMC8847732; doi:10.3389/fpsyg.2022.790494)
Supplement: Supplementary file 1 [file Data_Sheet_1.docx]

# Supplementary materials

## *Supplement 1.*

The question of the review and PICO

How does the implicit mentalization performance of patients with schizophrenia differ from that of the healthy controls?

P: General population

I: Schizophrenia

C: Healthy controls

O: Implicit mentalization performance

## *Supplement 2.*

Studies included from other sources

1. Das P, Lagopoulos J, Coulston CM, Henderson AF, Malhi GS: Mentalizing impairment in schizophrenia: a functional MRI study. Schizophr Res. 2012;134(2-3):158-164. doi:10.1016/j.schres.2011.08.019 *was cited by* Kronbichler L, Stelzig-schöler R, Pearce BG, Tschernegg M, Said-Yürekli S, Crone JS és mtsai: Reduced spontaneous perspective taking in schizophrenia. Psychiatry Res Neuroimaging. 2019; 292:5-12. doi:10.1016/j.pscychresns.2019.08.007
2. Eack SM, Wojtalik JA, Newhill CE, Keshavan MS, Phillips ML: Prefrontal cortical dysfunction during visual perspective-taking in schizophrenia. Schizophr Res. 2013;150(2-3):491-497. doi:10.1016/j.schres.2013.08.022 *was cited by* Kronbichler L, Stelzig-Schöler R, Pearce BG, Tschernegg M, Said-Yürekli S, Crone JS és mtsai: Reduced spontaneous perspective taking in schizophrenia. Psychiatry Res Neuroimaging. 2019; 292:5-12. doi:10.1016/j.pscychresns.2019.08.007
3. Veddum, L., Pedersen, H. L., Landert, A.-S. L., & Bliksted, V. (2019). Do patients with high-functioning autism have similar social cognitive deficits as patients with a chronic cause of schizophrenia? Nordic Journal of Psychiatry, 73(1), 44–50. <http://doi.org/10.1080/08039488.2018.1554697> (pooled record) *was cited by* Patel GH, Arkin SC, Ruiz-Betancourt DR, Debaun HM, Strauss NE, Bartel LP és mtsai: What you see is what you get: visual scanning failures of naturalistic social scenes in schizophrenia [published online ahead of print, 2020 Jun 5]. Psychol Med. 2020;1-10. doi:10.1017/S0033291720001646
4. Roux P, Forgeot D'arc B, Passerieux C, Ramus F.: Is the Theory of Mind deficit observed in visual paradigms in schizophrenia explained by an impaired attention toward gaze orientation?. Schizophr Res. 2014;157(1-3):78-83. doi:10.1016/j.schres.2014.04.031 *was cited by* Roux P, Smith P, Passerieux C, Ramus F.: Preserved implicit mentalizing in schizophrenia despite poor explicit performance: evidence from eye tracking. Sci Rep. 2016; 6:34728. Published 2016 Oct 5. doi:10.1038/srep34728
5. Roux P, Brunet-Gouet E, Passerieux C, Ramus F.: Eye-tracking reveals a slowdown of social context processing during intention attribution in patients with schizophrenia. J Psychiatry Neurosci. 2016;41(2): E13-E21. doi:10.1503/jpn.150045 *was cited by* Roux P, Smith P, Passerieux C, Ramus F.: Preserved implicit mentalizing in schizophrenia despite poor explicit performance: evidence from eye tracking. Sci Rep. 2016; 6:34728. Published (2016) 5. doi:10.1038/srep34728
6. Brunet, E., Sarfati, Y., Hardy-Bayle, M.C., Decety, J., 2003. Abnormalities of brain function during a nonverbal theory of mind task in schizophrenia. Neuropsychologia 41, 1574–1582. *was cited by* Das, P., et al., Mentalizing impairment in schizophrenia: A functional MRI study, Schizophr. Res. (2011) doi:10.1016/j.schres.2011.08.019
7. Roux P, Passerieux C, Ramus F. An eye-tracking investigation of intentional motion perception in patients with schizophrenia. J Psychiatry Neurosci 2015;40:118-25. *was cited by* Roux P, Brunet-Gouet E, Passerieux C, Ramus F.: Eye-tracking reveals a slowdown of social context processing during intention attribution in patients with schizophrenia. J Psychiatry Neurosci. 2016;41(2):E13-E21. doi:10.1503/jpn.150045
8. Okruszek, L., Wordecha, M., Jarkiewicz, M., Kossowski, B., Lee, J., & Marchewka, A. (2017). Brain correlates of recognition of communicative interactions from biological motion in schizophrenia. Psychological Medicine. Advance online publication. <http://dx.doi.org/10.1017/> S0033291717003385 *was cited by* Okruszek Ł, Piejka A, Wysokiński A, Szczepocka E, Manera V. Biological motion sensitivity, but not interpersonal predictive coding is impaired in schizophrenia. J Abnorm Psychol. 2018 Apr;127(3):305-313. doi: 10.1037/abn0000335. Epub 2018 Jan 25. PMID: 29369645.

## *Supplement 3.*

Characteristics of the 11 included studies.

|  | Design | Country | Centers |
| --- | --- | --- | --- |
| Brunet et al. 2003 | case-control | France | - |
| Das et al. 2012 | case-control | Australia | single |
| Eack et al. 2013 | case-control | USA | several |
| Kronbichler et al. 2019 | case-control | Austria | single |
| Okruszek et al. 2017 | case-control | Poland | several |
| Okruszek et al. 2018 | case-control | Poland | single |
| Patel et al. 2020 | case-control | USA | single |
| Roux et al. 2014 | case-control | France | several |
| Roux et al. 2015 | case-control | France | several |
| Roux et al. 2016 | case-control | France | several |
| Roux et al. 2016 | case-control | France | several |

*Supplement 4.* Risk of bias assessments

##
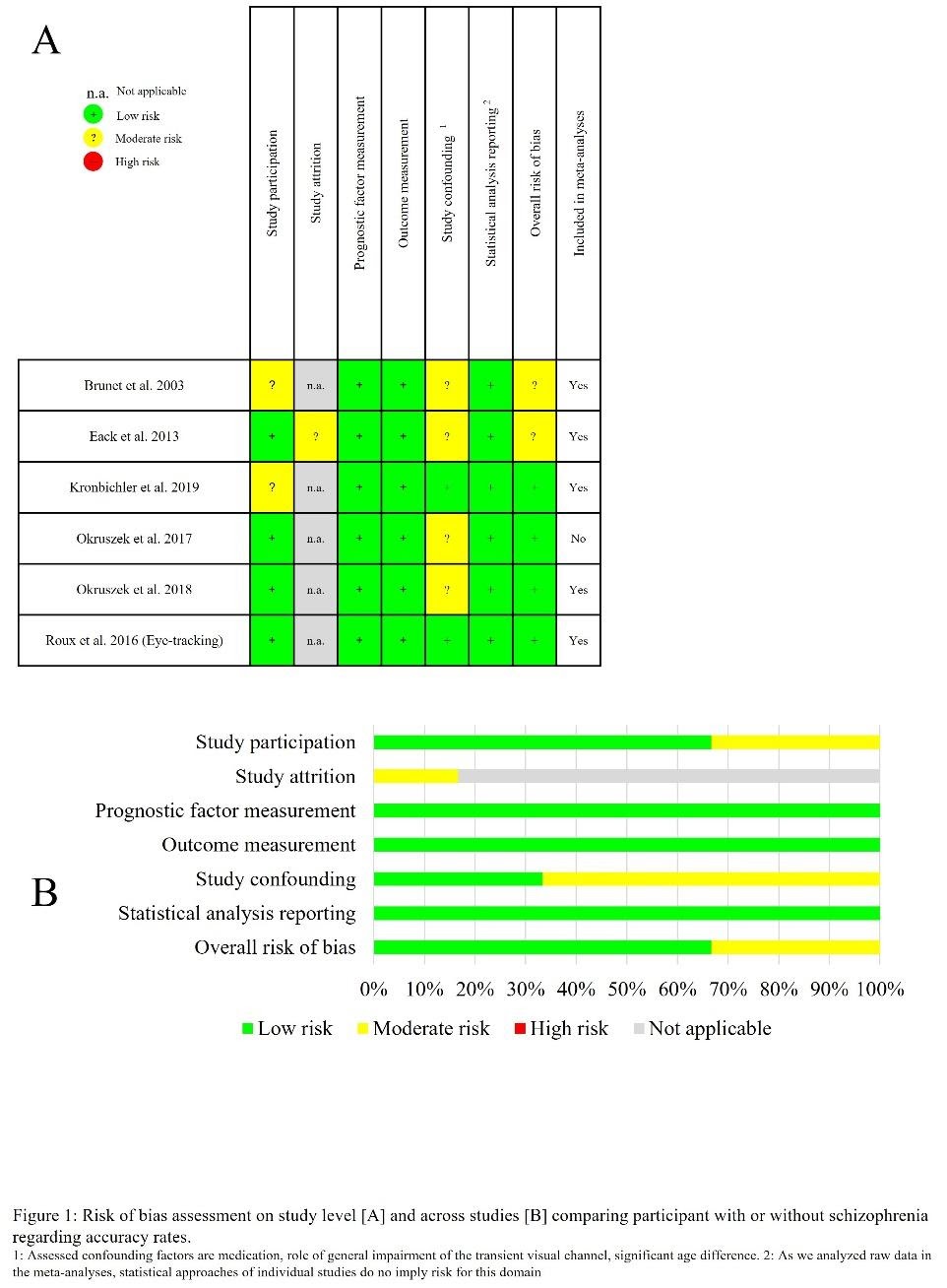

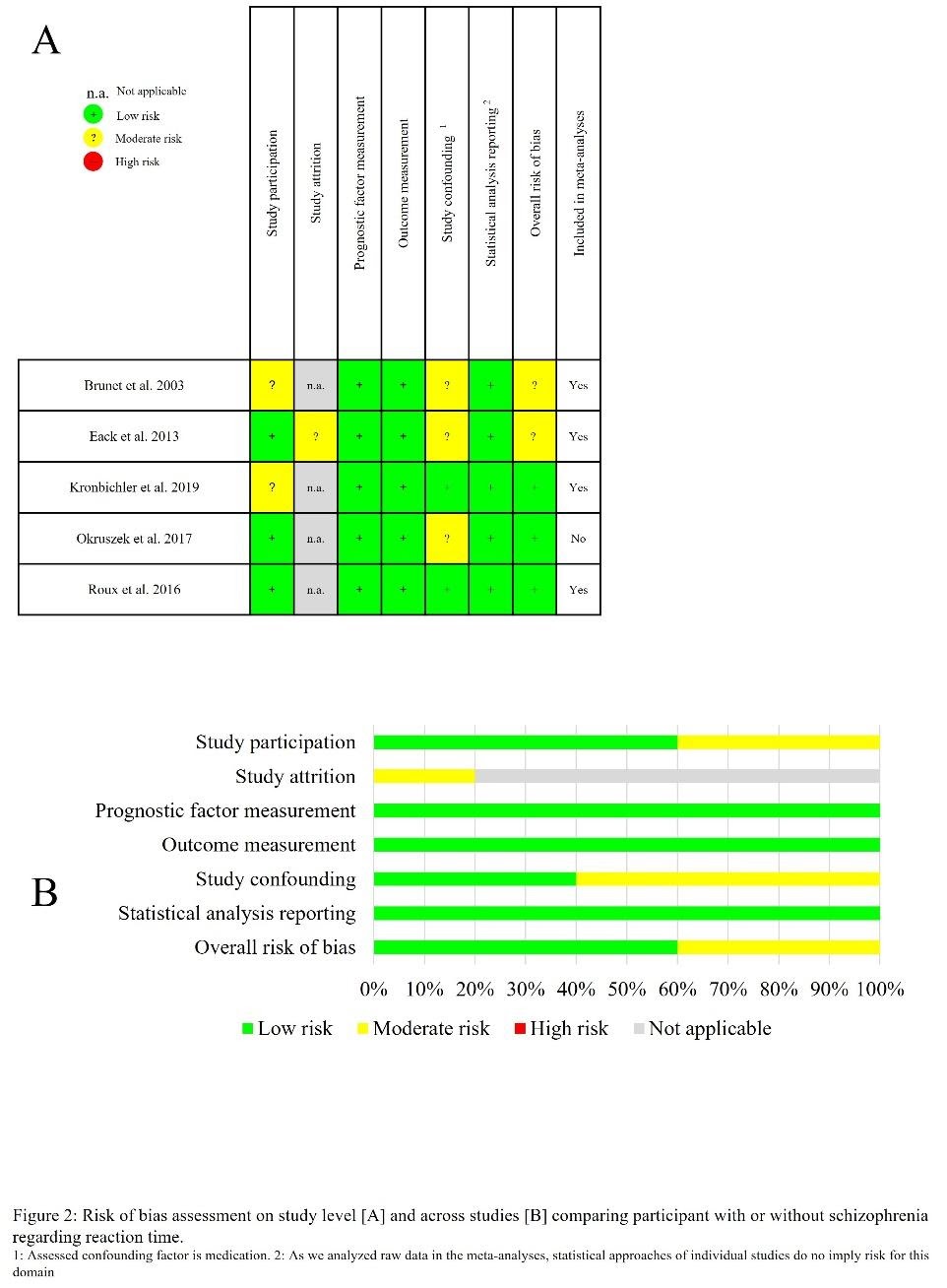


##
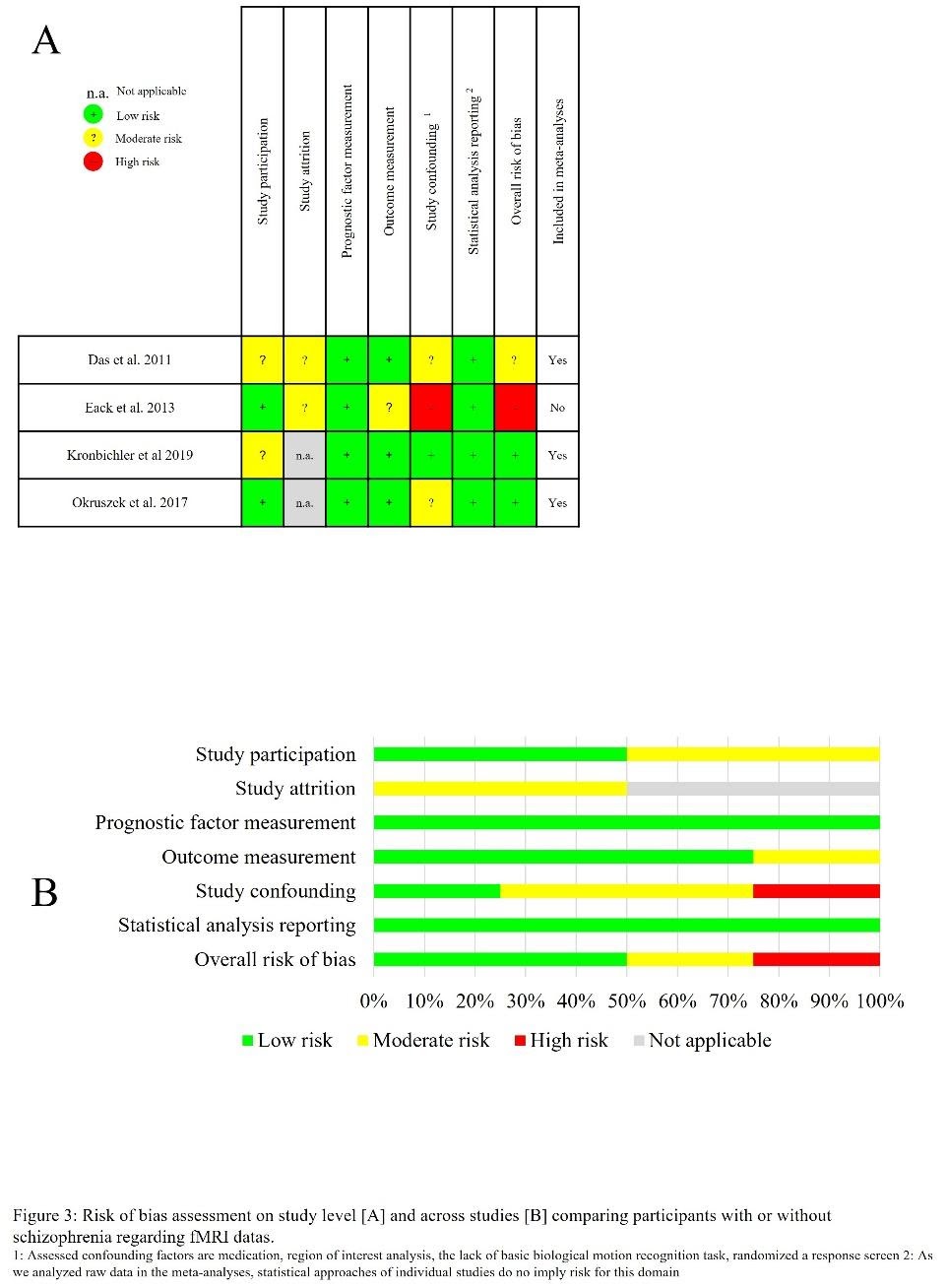

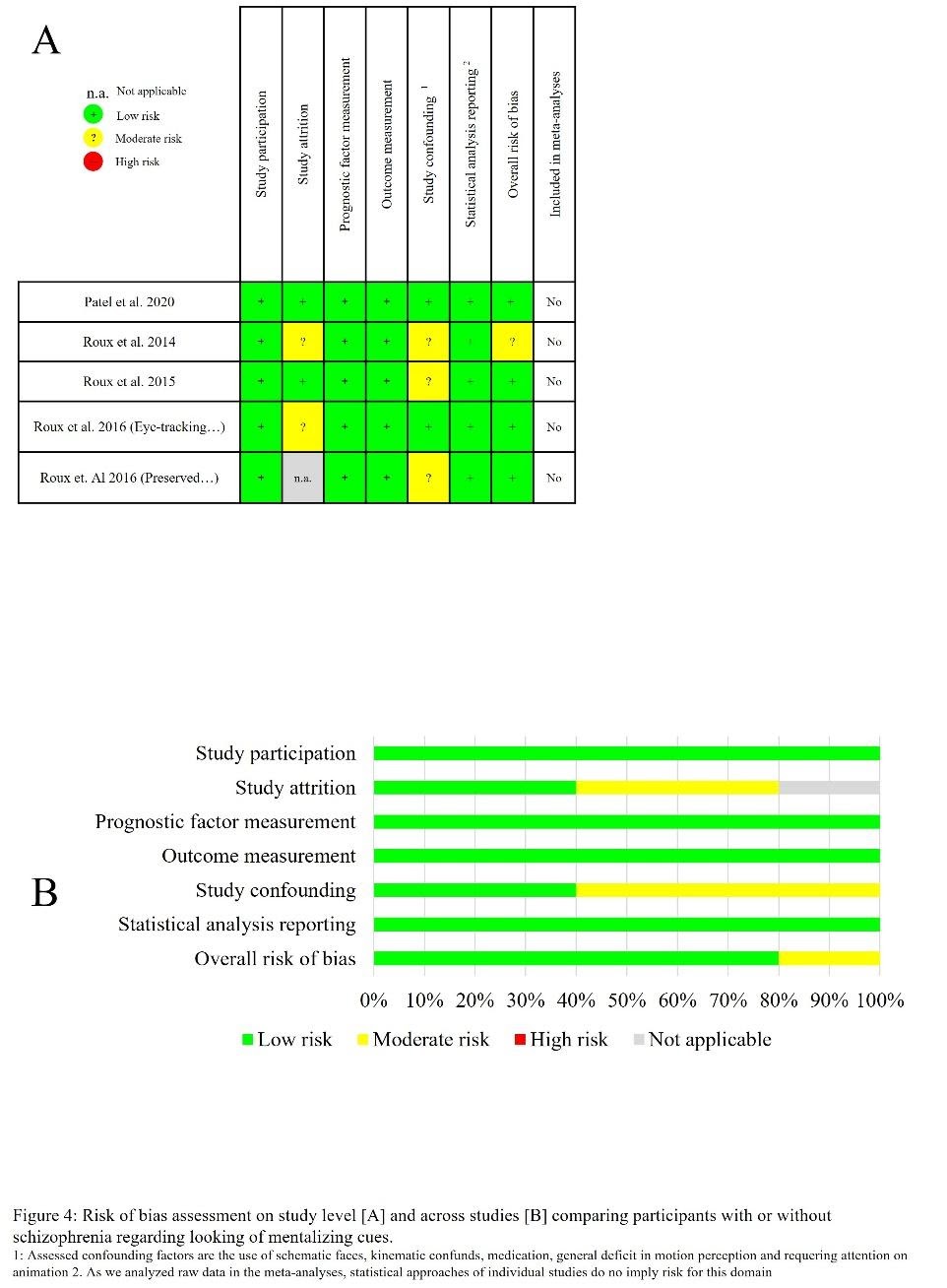


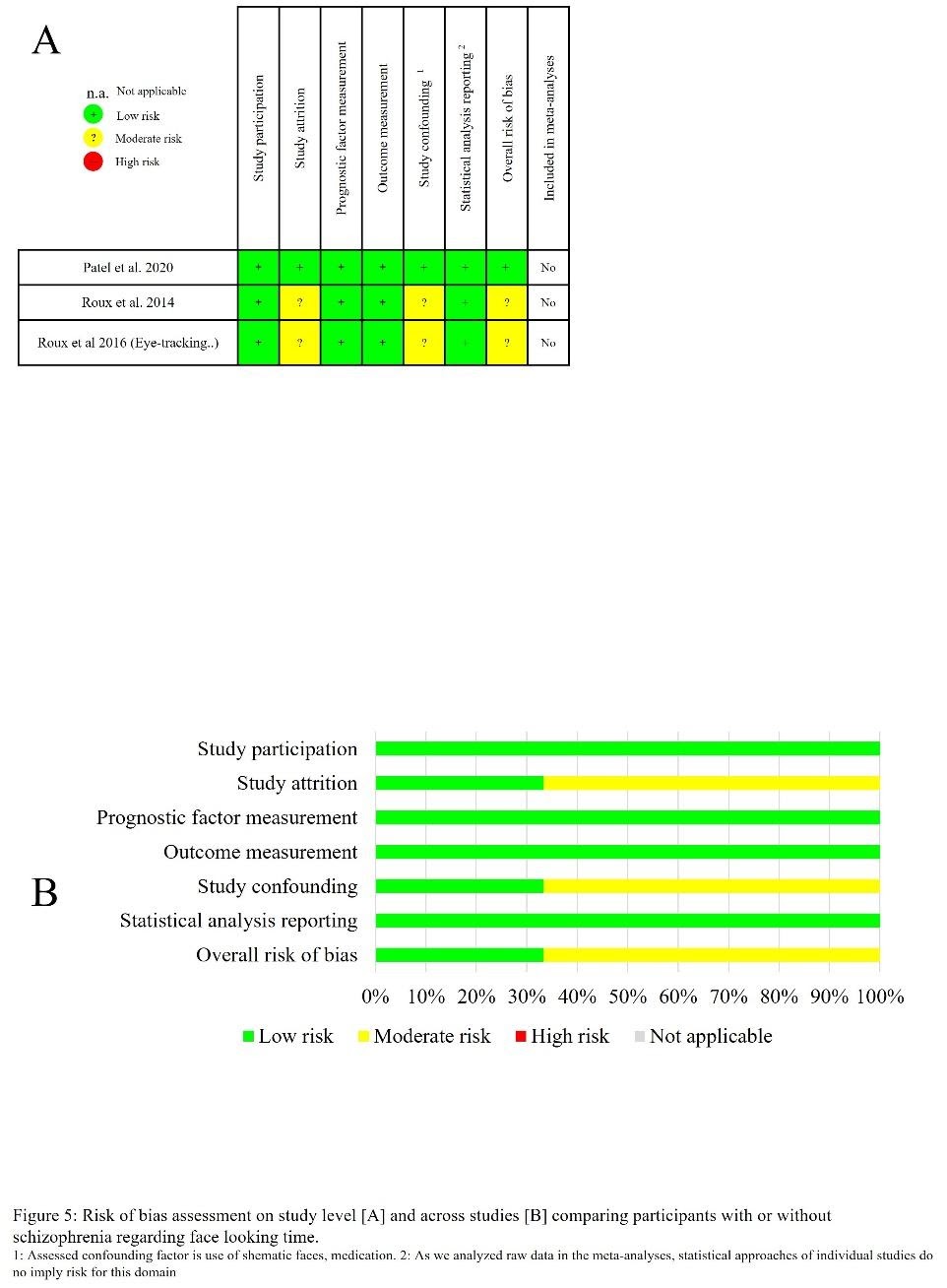
 
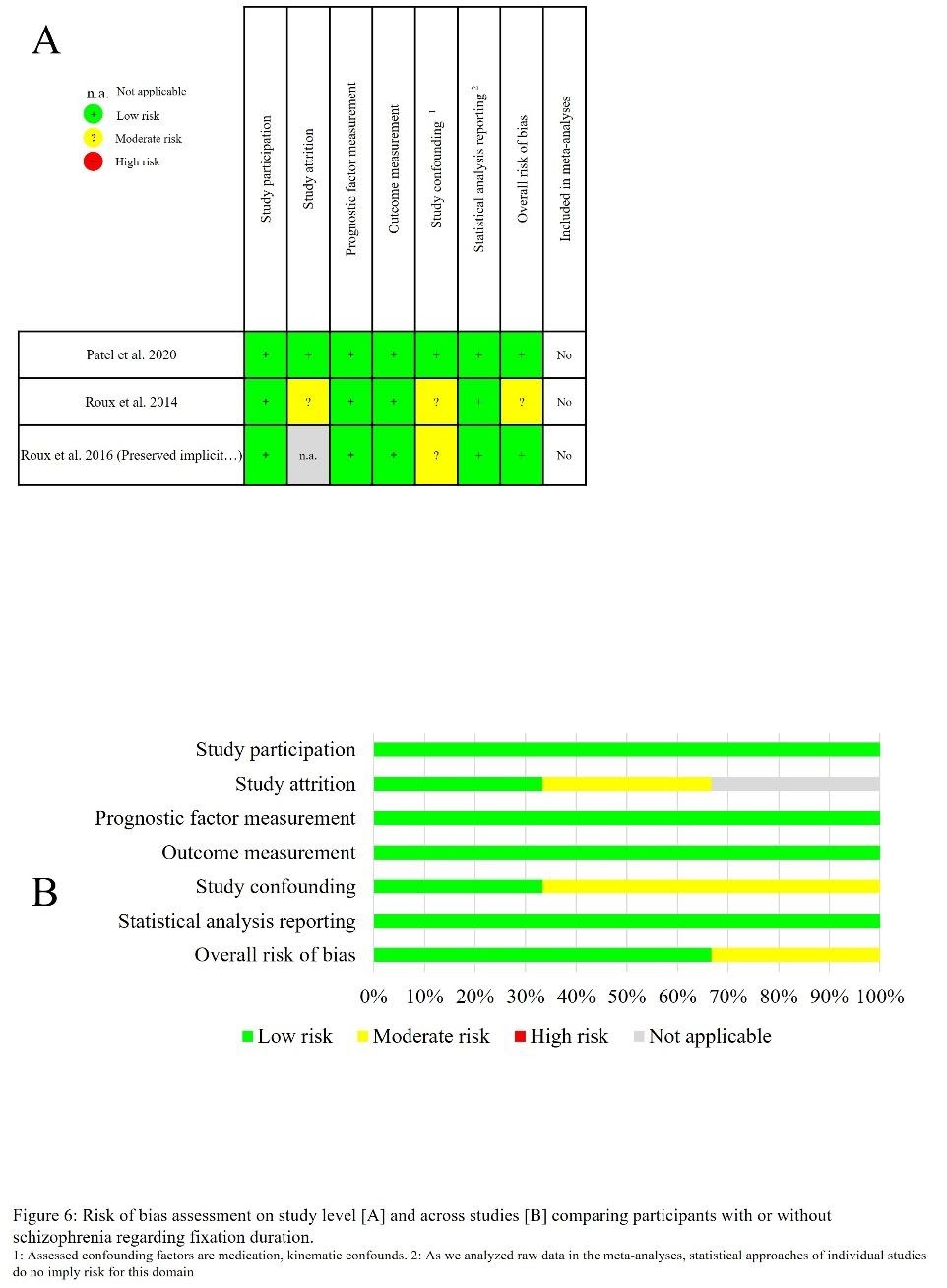


## *Supplement 5* PRISMA Checklist

| **Section and Topic** | **Item #** | **Checklist item** | **Location where item is reported** |
| --- | --- | --- | --- |
| **TITLE** | | |  |
| Title | 1 | Identify the report as a systematic review. | page 1 |
| **ABSTRACT** | | |  |
| Abstract | 2 | See the PRISMA 2020 for Abstracts checklist. | page 1 |
| **INTRODUCTION** | | |  |
| Rationale | 3 | Describe the rationale for the review in the context of existing knowledge. | page 2-5 |
| Objectives | 4 | Provide an explicit statement of the objective(s) or question(s) the review addresses. | page5 2 paragraph |
| **METHODS** | | |  |
| Eligibility criteria | 5 | Specify the inclusion and exclusion criteria for the review and how studies were grouped for the syntheses. | page6 4,5 p |
| Information sources | 6 | Specify all databases, registers, websites, organisations, reference lists and other sources searched or consulted to identify studies. Specify the date when each source was last searched or consulted. | page6 2 p |
| Search strategy | 7 | Present the full search strategies for all databases, registers and websites, including any filters and limits used. | page6 2p |
| Selection process | 8 | Specify the methods used to decide whether a study met the inclusion criteria of the review, including how many reviewers screened each record and each report retrieved, whether they worked independently, and if applicable, details of automation tools used in the process. | page6 4 p |
| Data collection process | 9 | Specify the methods used to collect data from reports, including how many reviewers collected data from each report, whether they worked independently, any processes for obtaining or confirming data from study investigators, and if applicable, details of automation tools used in the process. | page7 2p |
| Data items | 10a | List and define all outcomes for which data were sought. Specify whether all results that were compatible with each outcome domain in each study were sought (e.g. for all measures, time points, analyses), and if not, the methods used to decide which results to collect. | page7 2p |
|  | 10b | List and define all other variables for which data were sought (e.g. participant and intervention characteristics, funding sources). Describe any assumptions made about any missing or unclear information. | page7 2p |
| Study risk of bias assessment | 11 | Specify the methods used to assess risk of bias in the included studies, including details of the tool(s) used, how many reviewers assessed each study and whether they worked independently, and if applicable, details of automation tools used in the process. | page7 3p |
| Effect measures | 12 | Specify for each outcome the effect measure(s) (e.g. risk ratio, mean difference) used in the synthesis or presentation of results. | page7 5p |
| Synthesis methods | 13a | Describe the processes used to decide which studies were eligible for each synthesis (e.g. tabulating the study intervention characteristics and comparing against the planned groups for each synthesis (item #5)). | page6 4-5p |
|  | 13b | Describe any methods required to prepare the data for presentation or synthesis, such as handling of missing summary statistics, or data conversions. | page7 2p |
|  | 13c | Describe any methods used to tabulate or visually display results of individual studies and syntheses. | page7 2p |
|  | 13d | Describe any methods used to synthesize results and provide a rationale for the choice(s). If meta-analysis was performed, describe the model(s), method(s) to identify the presence and extent of statistical heterogeneity, and software package(s) used. | page7 5p |
|  | 13e | Describe any methods used to explore possible causes of heterogeneity among study results (e.g. subgroup analysis, meta-regression). | page19 1p |
|  | 13f | Describe any sensitivity analyses conducted to assess robustness of the synthesized results. | page7 5p |
| Reporting bias assessment | 14 | Describe any methods used to assess risk of bias due to missing results in a synthesis (arising from reporting biases). | page7 3p |
| Certainty assessment | 15 | Describe any methods used to assess certainty (or confidence) in the body of evidence for an outcome. | page8 4p |
| **RESULTS** | | |  |
| Study selection | 16a | Describe the results of the search and selection process, from the number of records identified in the search to the number of studies included in the review, ideally using a flow diagram. | page8 4p |
|  | 16b | Cite studies that might appear to meet the inclusion criteria, but which were excluded, and explain why they were excluded. | page8 4p  page32-34 |
| Study characteristics | 17 | Cite each included study and present its characteristics. | page10 |
| Risk of bias in studies | 18 | Present assessments of risk of bias for each included study. | page31-33 |
| Results of individual studies | 19 | For all outcomes, present, for each study: (a) summary statistics for each group (where appropriate) and (b) an effect estimate and its precision (e.g. confidence/credible interval), ideally using structured tables or plots. | page10-13 |
| Results of syntheses | 20a | For each synthesis, briefly summarise the characteristics and risk of bias among contributing studies. | page11-14 |
|  | 20b | Present results of all statistical syntheses conducted. If meta-analysis was done, present for each the summary estimate and its precision (e.g. confidence/credible interval) and measures of statistical heterogeneity. If comparing groups, describe the direction of the effect. | page11-14 |
|  | 20c | Present results of all investigations of possible causes of heterogeneity among study results. | page11; 19 2p |
|  | 20d | Present results of all sensitivity analyses conducted to assess the robustness of the synthesized results. | page 11-14 |
| Reporting biases | 21 | Present assessments of risk of bias due to missing results (arising from reporting biases) for each synthesis assessed. | page 31-33 |
| Certainty of evidence | 22 | Present assessments of certainty (or confidence) in the body of evidence for each outcome assessed. | page 13 1p |
| **DISCUSSION** | | |  |
| Discussion | 23a | Provide a general interpretation of the results in the context of other evidence. | page16-17 |
|  | 23b | Discuss any limitations of the evidence included in the review. | page18 3p; 19 1p |
|  | 23c | Discuss any limitations of the review processes used. | page18 3p; 19 1p |
|  | 23d | Discuss implications of the results for practice, policy, and future research. | page19 2p |
| **OTHER INFORMATION** | | |  |
| Registration and protocol | 24a | Provide registration information for the review, including register name and registration number, or state that the review was not registered. | page6 1p |
|  | 24b | Indicate where the review protocol can be accessed, or state that a protocol was not prepared. | page6 1p |
|  | 24c | Describe and explain any amendments to information provided at registration or in the protocol. | page6 1p |
| Support | 25 | Describe sources of financial or non-financial support for the review, and the role of the funders or sponsors in the review. | page20 1p |
| Competing interests | 26 | Declare any competing interests of review authors. | introductory page |
| Availability of data, code and other materials | 27 | Report which of the following are publicly available and where they can be found: template data collection forms; data extracted from included studies; data used for all analyses; analytic code; any other materials used in the review. | page 11-12 |

*From:*  Page MJ, McKenzie JE, Bossuyt PM, Boutron I, Hoffmann TC, Mulrow CD, et al. The PRISMA 2020 statement: an updated guideline for reporting systematic reviews. BMJ 2021;372:n71. doi: 10.1136/bmj.n71

For more information, visit: <http://www.prisma-statement.org/>
